# An assessment of the quality of the I-DSD and the I-CAH registries - international registries for rare conditions affecting sex development

**DOI:** 10.1186/s13023-017-0603-7

**Published:** 2017-03-20

**Authors:** M. Kourime, J. Bryce, J. Jiang, R. Nixon, M. Rodie, S.F. Ahmed

**Affiliations:** 1 Developmental Endocrinology Research Group, School of Medicine, University of Glasgow, Royal Hospital For Children, Office Block, 1345 Govan Road, Glasgow, G51 4TF UK; 2 School of Medicine and Pharmacy, University of Hassan II, Tarik Ibnou Ziad Road, Casablanca, 20250 Morocco

**Keywords:** Congenital adrenal hyperplasia, Data quality, Disorders of sex development, Research quality, Validity

## Abstract

**Background:**

With the proliferation of rare disease registries, there is a need for registries to undergo an assessment of their quality against agreed standards to ensure their long-term sustainability and acceptability.This study was performed to evaluate the I-DSD and I-CAH Registries and identify their strengths and weaknesses.

**Methods:**

The design and operational aspects of the registries were evaluated against published quality indicators. Additional criteria included the level of activity, international acceptability of the registries and their use for research.

**Results:**

The design of the I-DSD and I-CAH Registries provides them with the ability to perform multiple studies and meet the standards for data elements, data sources and eligibility criteria. The registries follow the standards for data security, governance, ethical and legal issues, sustainability and communication of activities. The data have a high degree of validity, consistency and accuracy and the completeness is maximal for specific conditions such as androgen insensitivity syndrome and congenital adrenal hyperplasia. In terms of research output, the external validity is strong but the wide variety of cases needs further review. The internal validity of data was condition specific and highest for conditions such as congenital adrenal hyperplasia. The shift of the registry from a European registry to an international registry and the creation of a discrete but linked CAH registry increased the number of users and stakeholders as well as the international acceptability of both registries.

**Conclusions:**

The I-DSD and I-CAH registries comply with the standards set by expert organisations. Recent modifications in their operation have allowed the registries to increase their user acceptability.

## Background

By enabling surveillance, audit and research through a virtual environment, registries have the potential to improve the care of people with rare conditions and diseases. These registries may be particularly useful for heterogeneous groups of rare conditions such as disorders of sex development (DSD) where the perceived stigma of the condition, the gaps in knowledge about aetiology and long-term outcome and the lack of expert and evidence-based multidisciplinary care result in substantial variation in patient care and disaffection [1]. In such situations, important lessons can only be learnt through the pooling of data within a common, secure platform and through effective interaction between researchers, health care professionals and the affected community. In the field of DSD, the impetus for this need for collaboration was set in motion following the consensus meeting a decade ago and has been sustained through further initiatives such as EuroDSD, I-DSD and DSDnet [2]. The registry infrastructure that has developed from these efforts initially involved a handful of centres in Europe but currently involves clinical users from all five continents. These health care professionals provide care for a range of conditions affecting sex development as well as congenital adrenal hyperplasia and the two registries, I-DSD and I-CAH, that have developed have now started to demonstrate their ability to perform several functions including research, patient management, patient accessible records and for clinical and expert networking.

As the I-DSD and I-CAH registries mature, the need for ensuring the quality and value of these registries becomes more important especially if there is a need to sustain them over the longer term. Until recently, there was little guidance around the criteria that could be assessed to explore the quality of a rare disease registry [3]. This study has been performed to describe the most important features of the I-DSD and I-CAH registries that allow them to be compared against quality benchmarks. In addition, the study also explores additional quality criteria that could be considered in the future.

## Methods

**Fig. 1:**
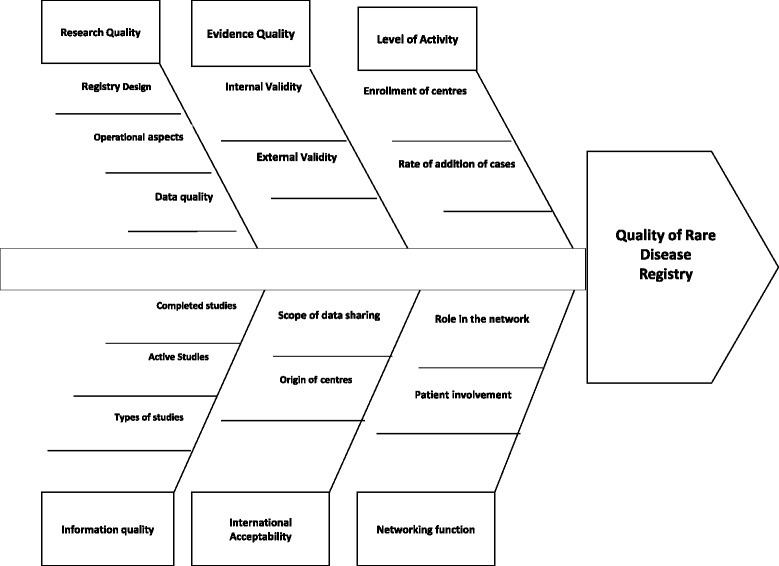
The assessment model of the I-DSD and I-CAH Registries

The evaluation of the quality of I-DSD and I-CAH Registries considered two domains; research quality and evidence quality [3]. Additional criteria were considered related to the level of activity, the international acceptability and the networking function of these Registries (Fig. 1).

### Research quality

Research quality was evaluated by assessing the design of the registries, their operational protocols and the quality of data. The design of the I-DSD and I-CAH registries was assessed according to criteria set by the Agency for Healthcare Research and Quality to protect against bias [3] (Table 1). The operational aspects of the Registry were compared to standards defined by the EPIRARE survey on a representative sample of 220 European rare disease registries [4]. The set of variables found to characterize high-quality RDRs according to this survey focused on ethical and legal issues, governance, communication of activities and results, established procedures to regulate access to data and security and established plans to ensure long-term sustainability (Table 2).

**Table 1:** Data quality indicators of Registry design that affect the quality of Research derived from the Registry according to the Agency for Healthcare Research and Quality

Parameter	Criteria of good Quality practice for Research
Purpose of the Registry	-Research questions clearly defined -Meet the needs of key stakeholders -The Registry should be an appropriate/the best means to achieve the purpose
Study Design	Choice of study design which is more efficient for addressing the research questions: Cohort, Case-control or case-cohort.
Data elements	Relevance to the objectives of the registry Acceptability to subjects and researchers^a^ Validity^b^ Reliability^c^ Use of standardized data collection form CDISC, CDASH, BRIDG when possible Use of disease coding system ICD coding system, MIM, ORPHA codes
Data sources (good quality means the use of the appropriate data sources to collect relevant data)	Clinician:accurate and specific clinical data Patient:data on health-related quality of life, utilities (patient preferences), behavioral data, family history. Electronic Health Record: information on routine medical care and practice, comprehensive view of patient medical and clinical history. Linkage with other sources:data difficult to obtain, subject to recall bias, not collected because of loss to followup, or likely inaccurate by self-report.
Population definition	- Patient selection: inclusion and exclusion criteriarelevant to the purpose - Patient sampling: consider representativeness in terms of patients and sites
Registry size and duration	Calculation of target sample size and definition of the duration of enrollment and follow-up should consider the aims of the registry, the desired precision of information sought, and the hypotheses to be tested.

*CDISC* Clinical Data Interchange Standards Consortium

*CDASH* Clinical Data Acquisition Standards Harmonization

*BRIDG* Biomedical Research Integrated Domain Group

^a^reasonably feasible to collect, minimal response burden

^b^does each data element truthfully measure what it is supposed to?

^c^can the instrument yield replicate metrics or estimates?

**Table 2:** Criteria of evaluation of quality of operational aspects [4]

Quality indicators	Profile of High Quality Rare Disease Registries
Ethical and Legal issues	-Protocols are approved by ethics committee. -Transparency of activities, oversight, data ownership and data delivery, and conflict of interest -Patient confidentiality, informed consent.
Access to data and security	-Data are made available anonymously to public institutions, public authorities, patient associations and private institutions/citizens, centres of expertise within the country and worldwide -Security is ensured by hosting data in a dedicated server and by an intrusion detection system. Having an approval by an external committee is required for accessing data
Communication of activities	Communication to data providers, public health policy makers and patient associations through websites, newsletters, institutional bulletins, scientific meetings and journals resulting in peer review by scientific journals and scientific meetings
Governance	A main governing board composed by internal and external experts has a good oversight and governance mechanisms dealing with financial, administrative, ethical and legal issues, research objectives, database content, data access and use, communication and coordination of all stakeholders.
Sustainability	Established plans to ensure durable funding and long-term sustainability
Data quality assurance procedures	-Case definition for the Rare Disease of interest -Standardized inclusion/exclusion -Data entered/sent (online/electronically) by data providers -Data periodically updated -Application of methods to avoid data entry mistakes -Use of quality indicators -Application of methods to check for reliability, agreement and internal validity -Periodic performance of quality tests/surveys -Application of methods to avoid duplication of registered cases -Availability of instructions for use of the registry -Provision of training or of a training kit for new users

The quality of data was assessed according to six primary factors set by the International Data Management Association and as summarized in Table 3 [5]. These data quality factors were assessed by analysing the data of the registry against itself, and by verification of all the case record except for timeliness and accuracy for which a subset of cases was used. The accuracy was difficult to assess because we needed to compare data in the registry with original data from centres which was not possible because of ethical and legal rules that protect data and prevent from identification of cases in the registry. The exercise was performed only for 23 PAIS cases where original data provided by centres were available in nine variables including phenotype evaluation by the EMS because of an ongoing study.

**Table 3:** The 6 Data Quality Dimensions defined by DAMA UK Working Group for data quality assessment

Data quality dimensions	Definition	Measure in the Registry
Completeness	The proportion of stored data against the potential of “100% complete”.	- Optional variables in Core data -All variables by disorders in all data -Optional variables by centresin all data, separately in the I-DSD and I-CAH Registries -Optional data in CAH longitudinal module
Uniqueness	No thing will be recorded more than once based upon how that thing is identified. Uniqueness is the inverse of an assessment of the level of duplication.	Percentage of duplicated cases by measuring data item against itself. A case is presumed duplicated when there is 100% similarity in core data and more than 90% similarity in non-core data between duplicates.
Timeliness	The degree to which data represent reality from the required point in time or how current or up to date the data are at the time of release.	Timeframe between the age at first presentation and the upload date in the Registry
Validity	Data are valid if it conforms to the syntax (format, type, range) of data definition.	The percentage of data that are not conform to the syntax in the longitudinal module in the ICAH Registry (Blood pressure)
Accuracy	The degree to which data correctly describes the “real world” object or event being described.	The accuracy of data in PAIS^a^ cases in the Registry was verified against original data available in templates completed by centres
Consistency	The absence of difference, when comparing two or more representations of a thing against a definition.	Consistency between the number of adverse events episodes^b^ and sick days in the longitudinal module of the I-CAH Registry

(When the data provider enter a number of adverse events, a table with a number of rows corresponding to the number of adverse event episodes is displayed and in each row we need to complete the number of sick days in each adverse event episode. The total number of sick days is automatically calculated. Obviously, the number of adverse events should not exceed the number of sick days, otherwise, there is an inconsistency between the two variables)

^a^ *PAIS* Partial androgen insensitivity syndrome

^b^ Adverse events: are the number of separate episodes of illness requiring extra dose of steroid

In terms of completeness, the core data were assessed separately as they represent the minimum mandatory requirement for cases to be eligible for entry in the registries. Further assessment of completeness of data was performed by diagnostic categories of conditions. An assessment of completeness of optional data fields also allowed a deeper insight into willingness of participating centres to provide complete data. The timeliness of data entry was evaluated in cases where the year at first presentation was 2008 or later as that was the year of the creation of the Registry. Only cases added by centres enrolled between 2008 and 2009 were included to avoid any bias introduced by the later enrolment of centres. The assessment of accuracy was performed by verifying a subset of data against original source data.

### External validity of data

**Fig. 2:**
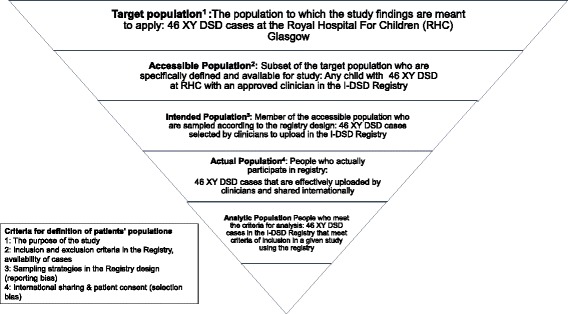
Description of the populations in the I-DSD Registry

The external validity of the registries was assessed by an evaluation of three broad criteria. Firstly, the representativeness of the actual population in the registries as assessed by both quantitative and semi-quantitative methods. The quantitative assessment compared the actual population to the target population in terms of absolute numbers. The studied actual population in the I-DSD Registry was 46 XY DSD cases assigned boys excluding CAH patients, uploaded by one centre, Glasgow, between 2010 and 2015 and shared internationally, because original source data were easily accessible in that centre for the same period (Fig. 2) The semi-quantitative assessment concentrated on the age distribution, the geographic distribution and the distribution of three conditions in the registries - CAH, AIS (Androgen Insensitivity Syndrome) and disorders of Müllerian development and was performed at two time points (2010 and 2016). This distribution was then compared with estimated incidence of these disorders according to published epidemiological data. The second criterion for assessing external validity was the completeness of information in the actual population and lastly, the third criterion that was assessed was case-attrition which was evaluated by calculating the number of patients or centres that had withdrawn from participation in the registries.

### Internal validity of data

The internal validity of the registries was assessed, firstly, by an evaluation of the extent of selection bias in the I-DSD registry evaluated by a study of the representativeness of cases described previously in Fig.1. In addition, internal validity was assessed by examining the information bias in the I-DSD Registry for cases of partial androgen insensitivity syndrome (PAIS) by assessing the discordance between a specific dataset relating to the external masculinisation score (EMS) at first presentation in the registry and EMS at first presentation in source data obtained by a separate template filled by clinicians for the same cases when participating in a previous study using the registry [6]. Lastly, internal validity was also assessed by studying the extent of misclassification of cases of XY DSD that had been classified as PAIS despite the absence of mutations in the androgen receptor gene.

### Level of activity & information quality

This was evaluated by the calculation of the rate of enrollment of centres in the Registry as network and clinical users, the progress of the percentage of centres adding new cases and the overall rate of addition of cases per year. The quality of research output was assessed by describing the number of completed studies and active studies using the registry and the number of users who were using the registries for research. The types of these studies were also evaluated.

### The international acceptability of the registries

This was assessed by calculating the rate of enrollment of non-European centres and the rate of addition of cases by these centres as well as the participation of non-European users in research using the Registry. The registries allow the clinical user to share their data according to four levels: international, European, national and local level only and the change in the level of sharing was assessed as a marker of internationalization of the registries.

### The professional networking function of the registries

This was evaluated by the calculation of the rate of enrollment of centres for networking purposes. The input of the project management group of the I-DSD Registry and I-CAH in other international networks (DSDnet, DSDlife, CAH-UK) and position statements was used as a marker of the networking function of the registries.

### Statistical analysis

Continuous variables were described as median and ranges and intergroup comparison for these variables was performed by Mann Whitney U tests. Chi-square test was used for categorical variables. P < .05 was considered to be statistically significant and all analyses were performed using SPSS for Windows software program, Version 22 (SPSS. Chicago, IL, USA).

## Results

### Measures for ensuring research quality

#### Design

The registries were developed to allow multiple studies using an observational study design based on a cohort of patients with DSD. The data elements could facilitate epidemiologic, genetic and clinical research. Only simple data that are routinely collected as part of clinical care are required allowing the participation of different centres. The original data elements were selected by a consensus around a case report form based on the revised DSD nomenclature [7]. Only clinicians approved by the Project Management Group and who are members of recognized professional societies can enter data in the Registry. The eligibility criteria for cases are broad as any adult or child with a condition associated with a DSD at a centre with an approved clinician can be included after obtaining consent. There are no restrictions regarding the number or characteristics of uploaded cases and this is left to self-selection by the clinician. As the registries were developed to allow multiple studies, the calculation of target sample size and follow up period is only undertaken when a new study is planned. The comparison groups are usually chosen from cases in the Registry. Centres are invited to participate in the new study, and they enter the required number of cases that fit the inclusion criteria for that study.

#### Operational protocols

The operational protocols for the registries have been approved by the UK NHS Information Governance Body, the UK Research Ethics Committee and the Ethics Committee of the former EuroDSD project funded by an EUFP7 grant. Ethical rules take into account the heterogeneity of legal structures of participant countries in terms of patient confidentiality, consent and regulations for biomaterial. The registries ensure data security by hosting data on a dedicated server and by using an intrusion detection system. Only clinical users and project management team can access the data that they have entered. Searching for data at centres other than the user’s centre is only possible via the project management team. In addition, a new option has been created to enable patients to access a portion of their record. The registries communicate their activities to a range of stakeholders including registry users, patients and their associations through regular presentations in national and international scientific meetings, via a website [8] and through 6-monthly newsletters. For governance, the Steering Committee is composed of clinical and non-clinical experts who provide the oversight and advise the project management group which coordinates the day-to-day activities including quality assurance of the registries. The registries were initially developed through funding received from the European Society for Paediatric Endocrinology, EUFP7 grant and the UK Medical Research Council. In the future, the sustainability of the registries will be dependent on project based funding and this has been demonstrated by its role in supporting new studies such as CAH-UK and PRO-CAH and its close links to the pharmaceutical industry.

#### Data quality

In order to ensure high quality data, the registries have implemented quality assurance procedures such as automated data cleaning which is performed by automated validation of data entry within a pre-defined range for some data fields such as weight, height, blood pressure, prevention of entry of alphabetic data in numeric fields (date, age), automated calculation of external masculinization score and deactivation of unnecessary data fields so that erroneous entries cannot occur. The data entry option ‘Yes’ ‘No’ ‘unknown’ and the option ‘Other’ in drop down lists allows the distinction between undocumented data and missing data and limited free text is included to add information not covered by the case report form. Description of some physical features such as the genitalia has been standardized by the use of Prader classification and EMS which are accompanied by visual cues as well as textual description. To avoid record duplication, each registered case is assigned a unique identifier associated with the local records at the data entry site. A comprehensive review of the data entered is generally only performed when the data are used by the biomedical community for research purposes. Instructions for the use of the Registry are available at the University of Glasgow website [9].

In terms of data completness, the results of assessment of this quality indicator  are available on Table 4. The median extent of completeness of optional data per case in the registries for DSD and CAH was 66% (4%-93%) and 52% (2%-87%) (*p* = 0.11). Of the 31 centres adding CAH cases, 15 (48%) had not only completed the cross-sectional module but were also completing the longitudinal module in 173 cases (30%). Of these 15 centres, 2 centres with an average of 40 CAH and 105 DSD cases each were consistently achieving a level of data completion of greater than 80%. Evidence of duplication using 68 variables was checked in 2155 cases and the number of duplicated cases was identified as 44 (2%). The timeliness of data entry was evaluated in 186 cases from 7 centres and the median time between age at first presentation and date of upload was 1 year (0-7). The validity of data entry was 100% when assessed for the blood pressure, the height and the weight in 314, 513 and 540 fields of 657 CAH visits respectively where data were available. Accuracy was checked in 23 cases of PAIS using 9 variables and an inaccuracy was found in 11 of the 207 fields (5%). In addition, inconsistency between the number of sick days and adverse events episodes was found in 28 of 165 visits (0.17%) where data were available.

**Table 4:** Completeness of data in the I-DSD and I-CAH Registries in core data, according to disorders and in optional data by centres

Data	Scope	Variables	Completeness <50%	Completeness 50–75%	Completeness ≥75%
Optional Core data	All cases in I-DSD and I-CAH Registries n1 = 2155	n = 2 -Actual diagnosis -Age of first presentation	-	-	99% 80%
All data	Data in each disorder in all cases in the I-DSD and I-CAH Registries n1 = 2155 (CAH Longitudinal module excluded)	n = 61	-Other (n1 = 144)	-Disorder of gonadal development (n1 = 418) Disorder of androgen synthesis (n1 = 247) -Non specific disorder of under masculisation (n1 = 209) -Persistant Müllerian Duct Syndrome (n1 = 15) -Cloacal anomaly (n1 = 4) -CAH (n1 = 604)	-Disorder of androgen action (n1 = 474) -Leydig Cell Defects (n1 = 23) -Defects of Müllerian development (n1 = 17)
Optional data	Data by centre in the I-DSD Registry n1 = 1551	n = 55	n3 = 17 n2 = 2 (1-224)	n3 = 9 n2 = 26 (6-91)	n3 = 20 n2 = 18 (1-122)
Optional data	Data by centre in the I-CAH Registry n1 = 604	n = 55	n3 = 14 n2 = 16 (1-54)	n3 = 10 n2 = 15 (5-59)	n3 = 7 n2 = 2 (1–26)
Optional data	Longitudinal module in the I-CAH Registry n1 = 173	n = 47	n3 = 2 n1 = 29	n3 = 2 n1 = 50	n3 = 11 n2 = 26 (4-72)

-Total Number of variables in the non longitudinal module; which is a common module between the two Registries; is 65 with 4 identifiers so, we assessed 61 variables with 6 variables which are mandatory to complete and 55 which are optional

-The core data mean the data that need completion in order to consider a case for a study. It contains the 4 identifiers, 3 mandatory variables (year of birth, Original Sex Assigned, Karyotype, and disorder type) and 2 optional variables (age at presentation and actual diagnosis)

-Total number of variables in the longitudinal module in the I-CAH Registry is 47

*n* number of variables

*n1* number of cases

*n2* median number of cases per centre (range)

*n3* number of centres

### External validity

**Fig. 3:**
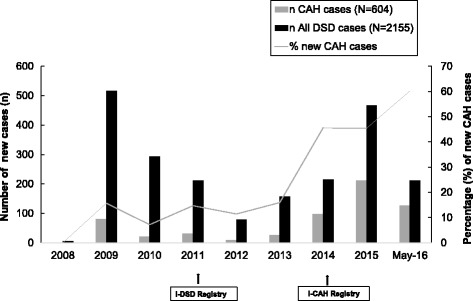
The rate of addition of new cases per year in the I-DSD and I-CAH Registries. n: Number of new cases added per year. N: Total number of cases in the Registry at the time of analysis

The quantitative assessment of representativeness shows that 69/122 cases (56.5% of the patients enrolled in the centre) are entered in the Registry and 53/122 cases (43.5%) are shared internationally. The median year of birth of 2155 cases entered in the registries was 2000 (1927-2016) with 1073 (50%) over 16 yrs. The median age at first presentation was 1-3 months (<1 month-63 years) with 149 from 1702 where information were available (8.7%) presenting over the age of 16 years. In 2009, user involvement was limited to 11 centres from 8 countries in Europe only. However, the percentage of cases added per year by non-European centres has increased significantly. Currently, cases have been entered from 55 centres from 26 countries from 5 continents. The majority of cases (1620;75%) are from 38 centres in 15 European countries and the majority of these are from the United Kingdom (491; 23%), Germany (275; 13%), Netherlands (264; 12%) and Italy (204; 9%). Of the 535 (25%) cases entered from 17 centres in 11 countries outside Europe, 268 (13%) are from Turkey and 132 (6%) from Egypt. There has been a shift in the proportion of cases with CAH that have been entered in the registries. The percentage of CAH cases added per year has increased by more than 6 fold (Fig. 3) and the percentage of centres adding these cases has followed the same pattern. In terms of distribution of conditions, the representativeness of the registry has increased. The proportion of cases of CAH compared to AIS (Androgen Insensitivity Syndrome) expressed by the CAH:AIS ratio has changed from 0.5 in February 2010 to 1.2 in May 2016 in the I-DSD Registry. This is approaching the estimated ratio in the population calculated by the estimated incidence of 1 in 15,000 for CAH [10] and of between 1:40,800 and 1:99,000 for AIS [11] with a CAH:AIS ratio between 2.5 and 6. However, other conditions that are associated with DSD such as disorders of Müllerian development (MRKH: Mayer-Rokitansky-Küster-Hauser syndrome) which have an incidence of MRKH in the population of 1 in 4,500 are underrepresented [12]. Thus the condition is 4.5 fold more frequent than AIS but the MRKH:AIS ratio in the registry was 0.02 and 0.03 respectively in Februray 2010 and in May 2016 . Concerning case-attrition, there was no withdrawal within participating centres in the registries and there have been no requests for deleting the records of any patients from any centres.

### Internal validity

It is likely that self-selection by clinicians of cases to upload into the registries and the consent process, itself, introduces a selection bias in the registries. This has not been explored systematically across all users. An assessment of information bias revealed that of 23 cases assessed, in 5 cases (22%), the EMS at first presentation was different between the data in the I-DSD registry and the source data. There were 249 cases in the I-DSD Registry with a PAIS-like phenotype. Of these, 211 (85%) were categorised as Disorder of Androgen action “PAIS”, 18 (7%) as Disorder of Androgen action “Other”and 20 (8%) as non-specific disorder of under masculinization, thus showing a measurable level of misclassification.

### Level of activity and information quality

**Fig. 4:**
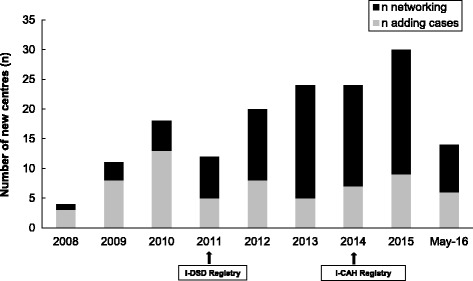
The rate of enrolment of new centres per year in the I-DSD and I-CAH Registries. n: Number of new centres enrolled per year

The rate of enrollment of centres has more than doubled between 2009 and 2015 with two clear peaks (Fig. 4). The first surge coincided with the move from EuroDSD Registry to I-DSD Registry. The second surge coincided with the creation of a dedicated I-CAH Registry. The rate of addition of cases has followed the same pattern (Fig. 3).

Completed studies using the Registry have led to six publications and 25 presentations at international scientific meetings. Currently, there are 12 additional active studies and 9 submitted for approval. Of the 279 Registry users, 65 (23%) have used the Registry for research, with a median number of studies per user of 2 [1, 11]. Of the 65, 18 (30%) of users are from outside Europe. The type of studies range from basic studies aimed at characterizing the DSD population [13] to those exploring trends in practice [14, 15] and novel associations [16] as well as frequency of adverse events [6, 17] The research output of these studies contributes to better outcome of patients. The study investigating novel associations has demonstrated that the rate of associated conditions is 10 times the birth prevalence of congenital anomalies [16] highlighting the need for input from multiple specialists with a multidisciplinary model of care. The poorer long term outcome of cases with confirmed PAIS compared to cases without Androgen Receptor mutation [6] emphasizes the necessity of AR analysis early in order to personalize care. The study investigating adverse events will contribute to better management of CAH patients with better definition of optimal doses of replacement therapy [17]. Other studies included the assessment of the practice of the users of the registries [18].

### International acceptability of the registries for research & networking

**Fig. 5:**
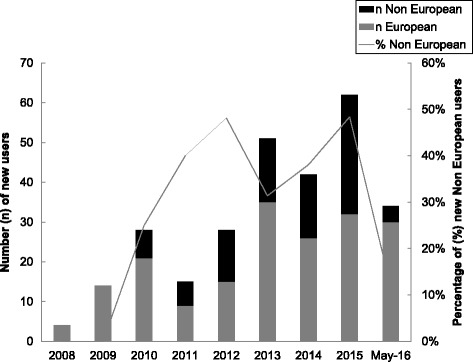
The rate of enrolment of European and non-European users in the I-DSD and I-CAH Registries. n: Number of new users. %: Percentage of new non European users

**Fig. 6:**
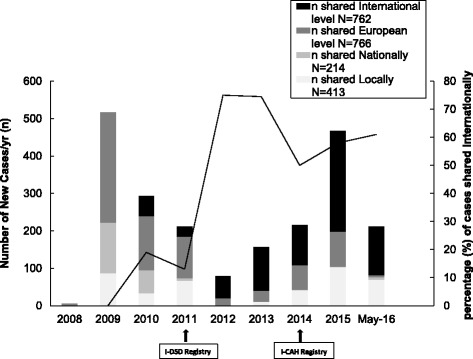
The evolution of the level of data sharing in the I-DSD and I-CAH Registries. n: Number of new cases per year shared at a certain level (international, national….). N: Total Number of cases in the Registry shared at that level at the time of analysis

The proportion of non-European users enrolling annually has doubled from only 25% in 2010 to 50% of new users in 2015 (Fig. 5). In addition to uploading details of cases, there has also been an increase in the rate of enrollment of centres for networking purposes (Fig. 4). Of the 279 users, 188 (67%) are from Europe and of these 188 registered users, 47 (25%) are involved in performing research. Of the 91 registered users from outside Europe, 18 (20%) are using the registries for performing research. Furthermore, with the involvement of more centres beyond Europe, the scope of data sharing has also increased so that more data are shared internationally rather than just simply within Europe (Fig. 6). This observation also applies to centres within Europe. Currently, 47 (86%) centres are sharing data internationally and only 2 (4%) are sharing data locally only. The networking function of the registries has allowed the registry users to play leading roles in initiatives such as EuroDSD [19], DSDlife [20] EU COST Action DSDnet [21] the recent global DSD update [22] the response to the EU Commissioner [23], a survey of DSD centres [18], a survey of clinical psychology support at DSD centres, and a biennial international meeting for DSD. The patient engagement in the registry demonstrates the acceptability in the patient community. Patient support groups are actively involved in the steering committee and participate in all decisions concerning the governance of the registry. As part of a COST Action, DSDnet, a workshop for patients and parents was organized in Bologna in October 2016 to identify the needs of patients from the registry, their preferences in terms of consent and access to data and research priorities. A web-based module within the registry has also been created enabling patients to view their data and to stay informed about active studies and express their preferences in healthcare and research.

## Discussion

According to Orphanet, there are over 650 registries for rare conditions [24]. With the proliferation of registries for rare conditions, it is imperative that these valuable resources undergo a quality assurance check so that they can be sustained for longer. The current study represents a comprehensive assessment of the quality of such a registry against standards that were recently suggested [3, 4].

Desirable qualities of disease registries include a comprehensive and collaborative approach to patient data collection that can ultimately address the requirements of patients, researchers and regulatory agencies [3, 25]. The design of the Registries meet the standards set in order to optimize their output in terms of the clarity of the purposes which consider all stakeholders perspectives, the study design and the broad inclusion criteria that allow multiple studies and generalisability of results, the entry of data by experienced specialized clinicians which contributes to data quality. The inclusion of patient reported data in the near future along with the use of a coding system will enhance further the quality of the design.

In terms of operational aspects of registries, high quality registries are prioritizing ethical and legal standards and are expected to provide access to data on a platform that ensures data security and patient confidentiality. Registries should ensure that they employ communication strategies that allow the dissemination of research activities and their results, thus promoting wider involvement of the stakeholders and ensuring adaptability and sustainability. Of the 272 registries surveyed by EPIRARE, 57% were not patient-based, 48% did not have a clear strategy for long-term sustainability, 34% did not have a clear management group, 30% did not share data, 21% were established without any clear funding and only 18% were truly international [26]. The I-DSD and I-CAH registries have met these benchmarks through a strong governance model which reflects both expertise and representation of several stakeholders including patients at all levels of their development, management and maintenance. The Registry has considerable acceptability in the patient community as demonstrated by a survey of patients where more than 90% of the respondents wanted to use the registry to access information regarding diagnosis, surgery, investigations, genetics and medication [27].

Participation in research and ensuring a regular research output is vital for a thriving registry. Not only have the I-DSD and I-CAH registries been used for performing research but they have also shown their ability to facilitate the development of a professional network. By using the registries to perform surveys of specialist health care provision [18] and by supporting a biennial international conference, the registries have attempted to become an integral tool for professional clinical networking as well as research collaboration.

In terms of data quality, the two registries that we studied exhibit, both, strengths and opportunities for improvement. As expected, the completeness of the data was higher for conditions such as disorders of androgen action and congenital adrenal hyperplasia and this may reflect the current focus of research [6, 14–17] Whilst, it is possible that this may introduce a level of selection bias and limit future studies to these specific conditions, it was interesting to note that centres that achieved a high level of completeness of data for these conditions also showed a high level of data completion for a wider range of conditions included within the registries. Thus, it seems that participation of centres in studies is related to greater and wider involvement that extends to other conditions within the registry that are beyond the areas of current focus. These active centres can act as the exemplars for the future as they adhere to one of the recommended quality criteria for centres of expertise for rare conditions that stipulates that such centres should participate in registries for rare conditions. Whilst it is possible that many more centres collect these data in local registries [18] for rare conditions, full participation in international registries has to be considered the gold standard for centres of expertise that are truly interested in driving the extent of research and quality of care [25]. Not only does a high level of participation increase the potential for meaningful research but it also increases the external validity of registries themselves. Although the external validity of the I-DSD and I-CAH registries was considered to be high when considering the heterogeneity of the population in terms of the distribution of age, geography and ethnicities as well as the representativeness of centres with different practices, the selection bias in cases included in the registries will need further attention. The high level of external validity of these registries is also illustrated by the relative ease with which the original I-DSD registry was used as a platform to develop the I-CAH registry.

The estimation of duplicated cases in the current quality assurance exercise was assessed to be low and similar to that reported by other registries that checked duplicates against the original source data [28]. Although the I-DSD and I-CAH Registries are not truly epidemiological registries, they have been successfully used to show temporal trends in practice [14, 15] and the prevalence of associated conditions [16]. However, the assessment of timeliness of data entry showed that a period of one year is possible and if this period of data entry was possible across all participating centres, then the registries would reach the level of timeliness that is commonly required by other registries such as the ones in the field of childhood cancer that regularly report annual incidence [29]. Assessing the accuracy of data in an international registry can be challenging due to restrictions imposed by geography as well as rules on confidentiality. To maximise the accuracy of the data as well as minimise the burden on the reporting clinician, the registries have limited the mandatory set of required data fields entry to a minimum. There is also a large concern on the accuracy of phenotype description due to variability of report between users. Measures are also undertaken in order to reduce this variability; a data dictionary is included in the assessment module as well as images in order to make the descriptions more uniform between data providers. Charts for penile and Clitoral length reference range are also included. The EMS is automatically calculated after data entry in order to minimize the risk of error. In addition, the registries have relied on checking accuracy by participation in specific studies and this exercise showed that the current rate of inaccuracy of 5% is comparable to that reported for other long-standing established registries [28, 30, 31]. This rate of inaccuracy could be considered as low and acceptable if it is random rather than systematic. However, an assessment of this will require the registries to explore other strategies to check inaccuracy such as random sampling of cases. It is also possible that a greater level of scrutiny of their personal data by the patients themselves may also lead to an improvement in data quality. The internal validity of the registries was considered to be strong when considering the structure of data elements in the I-CAH Registry that collected all factors that contribute to the outcome as well as potential confounders. However, it is possible that the information bias and misclassification can be reduced further by the use of data dictionary and training in data collection procedures.

Historically, the lack of a suitable ontology for rare conditions within commonly-used classifications of diseases, such as ICD, was one of the key drivers for the development of registries for rare conditions. In the field of DSD, this need was particularly apparent following the creation of the new nomenclature in 2005 [7]. Although the incorporation of this nomenclature in the I-DSD and I-CAH registries has allowed rapid acceptance of the registries by its core clinical users, the unique classification may, on the other hand, limits its interoperability with other registries for rare conditions. Around 25% of cases of DSD may have an anomaly in a system other than sex development [16]. An ability for the registries to map to Orphanet codes [32] as well as future versions of ICD that include more rare conditions [33] will need further exploration. With the increasing number of registries for rare conditions there is an increased likelihood that the same patient may be included in more than one registry and there is also the need to consider cross-talk between registries. In addition to a global unique identifier, it is likely that the inclusion of common data elements in the I-DSD and I-CAH registries as suggested by EUCERD, would allow greater interoperability and facilitate compliance with the guiding principles for scientific data management [34, 35]. More recently, the I-DSD and I-CAH registries have started to offer patients access to their data through a web-based portal. This is a facet to these registries which will be developed further over the coming years so that the registries can collect patient reported outcome data.

## Conclusion

An assessment of the quality of the I-DSD and I-CAH registries against recent standards set for registries for rare conditions has demonstrated a high level of compliance with these standards. The assessment has also revealed some deficiencies which will be addressed as the project develops further. Registries for rare conditions should routinely undergo a quality assessment exercise.

## Abbreviations

AIS: Androgen insensitivity syndrome
CAH: Congenital adrenal hyperplasia
DSD: Disorders of sex development
EMS: External masculinisation score
EPIRARE: European platform for rare diseases registries
EUCERD: European union committee of experts on rare diseases
EUFP7: European union framework program 7
I-CAH Registry: International congenital hyperplasia registry
ICD: International coding system
I-DSD Registry: International disorders of sex development registry
MRKH: Mayer-Rokitansky-Kuster-Hauser syndrome
PAIS: Partial androgen insensitivity syndrome
